# Correction: Lv et al. Investigation on Preparation and Performance of High Ga CIGS Absorbers and Their Solar Cells. *Materials* 2023, *16*, 2806

**DOI:** 10.3390/ma19091724

**Published:** 2026-04-24

**Authors:** Xiaoyu Lv, Zilong Zheng, Ming Zhao, Hanpeng Wang, Daming Zhuang

**Affiliations:** 1 Faculty of Materials and Manufacturing, Beijing University of Technology, Beijing 100124, China; lxy4518@emails.bjut.edu.cn (X.L.); zilong.zheng@bjut.edu.cn (Z.Z.); 2 School of Materials Science and Engineering, Tsinghua University, Beijing 100084, China; zhaoming2013@tsinghua.edu.cn (M.Z.); wanghp20@mails.tsinghua.edu.cn (H.W.)

The journal’s Editorial Office and Editorial Board are jointly issuing a resolution and removal of the Journal Notice linked to this article [1], as well as an update to the original publication. Following concerns raised about the integrity of the peer review, the Editorial Office has conducted a post-publication peer review of this article. This process included the recruitment of a new independent reviewer, and it was supervised by the original Academic Editors and an Editorial Board member to ensure full compliance with MDPI’s Editorial Process (https://www.mdpi.com/editorial_process)

As a result of this process, the original Academic Editors, the Editorial Board member, and the authors have agreed to update the following aspects of this publication:

- Reviewer reports 2 and 3 were removed from the peer review record, and the new report was uploaded to the peer review record (https://www.mdpi.com/1996-1944/16/7/2806/review_report).
- There was an update to the Academic Editors.
- The Editorial Board member who conducted this post-publication review has been added.
- In Section 1., the Introduction, Paragraphs 1 and 2 have been simplified.
- In Section 3.1.1., Cu(In,Ga)Se_2_ Growth and Characterization, Paragraph 2, the word “planeness” has been changed to “flatness”.
- The formatting of Figure 1 has been updated to improve readability.
- In Section 3.1.2., Characterization of CIGS Solar Cells, Paragraph 3, the sentence “Therefore, compared with the solar cell prepared by evaporation [18], the solar cell in this work has a higher Jsc at the same GGI, which may enable it to perform better in the tandem cells.” has been added to the end of this paragraph.
- Removed the following irrelevant references [1–3,8,18]. With this correction, the order of some references has been adjusted accordingly.

1. Zeenat; Maryum Javed, S.; Ahmad, Z.; Ahmed, S.; Iqbal, S.; Naqvi, I.J.; Usman, M.; Ashiq, M.N.; Elnaggar, A.Y.; El-Bahy, Z.M. Highly dispersed active sites of Ni nanoparticles onto hierarchical reduced graphene oxide architecture towards efficient water oxidation. *Fuel* **2022**, *312*, 122926.
2. Kuang, C.; Tan, P.; Javed, M.; Humaira Khushi, H.; Nadeem, S.; Iqbal, S.; Alshammari, F.H.; Alqahtani, M.D.; Alsaab, H.O.; Awwad, N.S.; et al. Boosting photocatalytic interaction of sulphur doped reduced graphene oxide-based S@rGO/NiS_2_ nanocomposite for destruction of pathogens and organic pollutant degradation caused by visible light. *Inorg. Chem. Commun*. **2022**, *141*, 109575.
3. Li, H.; Ji, H.; Liu, J.; Liu, W.; Li, F.; Shen, Z. Interfacial modulation of ZnIn_2_S_4_ with high active Zr-S_4_ sites for boosting photocatalytic activation of oxygen and degradation of emerging contaminant. *Appl. Catal. B Environ*. **2023**, *328*, 122481.
8. Nakamura, M.; Yamaguchi, K.; Kimoto, Y.; Yasaki, Y.; Kato, T.; Sugimoto, H. Cd-Free Cu(In,Ga)(Se,S)_2_ Thin-Film Solar Cell with Record Efficiency of 23.35%. *IEEE J. Photovolt.* **2019**, *9*, 1863–1867.
18. Dos Santos, R.B.; Rivelino, R.; Mota Fde, B.; Kakanakova-Georgieva, A.; Gueorguiev, G.K. Feasibility of novel (H_3_C)_n_X(SiH_3_)_3−n_ compounds (X = B, Al, Ga, In): Structure, stability, reactivity, and Raman characterization from ab initio calculations. *Dalton Trans.* **2015**, *44*, 3356–3366.

With this update, the Academic Editors are satisfied that the Editorial Process relating to this article has been completed as per MDPI’s Editorial Process policy. The Editorial Office would like to thank the authors for their collaboration during this process.
